# Noncoding RNAs in Steroid-Induced Osteonecrosis of the Femoral Head

**DOI:** 10.1155/2019/8140595

**Published:** 2019-12-23

**Authors:** Xinjie Wu, Wei Sun, Mingsheng Tan

**Affiliations:** ^1^Peking University China-Japan Friendship School of Clinical Medicine, Beijing 100029, China; ^2^Department of Orthopedic Surgery, China-Japan Friendship Hospital, Beijing 100029, China

## Abstract

Steroid-induced osteonecrosis of the femoral head (ONFH) is a severe orthopedic disease caused by the long-term administration of glucocorticoids. The main pathological feature of ONFH is the gradually progressive necrosis of bone cells and the bone marrow, ultimately resulting in structural changes or even complete collapse of the femoral head. However, the exact pathogenic mechanism of ONFH remains unknown. Noncoding RNAs (ncRNAs) have emerged as very powerful regulators of gene expression, functioning at both transcriptional and posttranscriptional levels in the pathogenesis of ONFH. Here, we review the current knowledge of the role of ncRNAs, including microRNAs, long noncoding RNAs, and circular RNAs, in the pathogenesis of steroid-induced ONFH. Further focus and validation of these associations can provide new insight into the pathogenic mechanisms at the molecular level to suggest targets for treatment and prevention.

## 1. Introduction

Osteonecrosis of the femoral head (ONFH) is characterized by the progressive necrosis of bone cells and the bone marrow, with an estimated incidence of 300,000–600,000 cases in the general population of the United States in the early 2000s [1]. The incidence of newly diagnosed cases of ONFH has remained stable at approximately 20,000 to 30,000 per year [2]. According to the etiology, ONFH can be categorized as traumatic and nontraumatic. Steroid administration and diseases requiring treatment with steroids are the main causes of nontraumatic ONFH, representing 30–50% of all cases of ONFH, mainly affecting adults aged 30–60 years in China [3]. Steroid-induced ONFH is a complex pathological process in which a variety of internal and external factors lead to intramedullary microvascular lesions, and the thrombosis causes insufficient blood and oxygen supply to the femoral head, resulting in osteocyte death. However, the precise pathogenesis and molecular mechanisms contributing to disease onset remain unclear. As it is clinically unfeasible to perform biopsies for diagnosing steroid-induced ONFH, biomarkers for early diagnosis are urgently needed.

Notably, only about 1.5% of the human genome comprises protein-coding regions, while the remaining sequences represent transcripts with no protein-coding capacity, collectively known as noncoding RNAs (ncRNAs) [4]. Although originally considered “noise” or “junk” RNAs, ncRNAs are now widely recognized as important regulators of gene expression, functioning at both transcriptional and posttranscriptional levels. Although the roles of ncRNAs in the development and progression of ONFH are gradually coming to light with progress in high-throughput sequencing techniques and associated analyses, there has been no comprehensive assessment of the related literature [5, 6]. Accordingly, we here provide an overview on the role of ncRNAs in steroid-induced ONFH pathogenesis.

## 2. MicroRNAs in Steroid-Induced ONFH

MicroRNAs (miRNAs) are a group of small-fragment, single-stranded, endogenous noncoding RNAs that negatively modulate the expression of target mRNAs mostly through binding to their 3′-untranslated region (3′-UTR) to regulate their translation and/or stability. Previous studies have shown that miRNAs play important roles in a variety of physiological processes, including cell development, proliferation, differentiation, metabolism, migration, and apoptosis [7, 8]. According to a bioinformatics analysis, more than one-third of all human genes are estimated to be regulated by miRNAs, indicating their important roles in regulating gene expression [9]. The miRNA expression profiles and functions in steroid-induced ONFH are summarized in Tables 1 and 2, respectively.

### 2.1. miRNA Expression Profiles in ONFH Tissues

Li et al. [17] found a subset of miRNAs that were differentially expressed by more than two-fold in the collapse area compared to the noncollapse area in three patients with steroid-induced ONFH. Specifically, eight miRNAs were found to be upregulated (hsa-miR-4472, hsa-miR-4306, hsa-miR-4747-5p, hsa-miR-4441, hsa-miR-4709-3p, ebv-miR-BHRF1-2-3p, hsa-miR-585-3p, and hsa-miR-5572) and two miRNAs were downregulated (hsa-miR-195-5p and hsa-miR-645). Compared to the normal tissue region, the most significantly downregulated miRNA, has-miR-195-5p, was confirmed by reverse transcription-quantitative polymerase chain reaction (RT-qPCR). Yuan et al. [18] identified 28 miRNAs exhibiting more than three-fold changes in steroid-induced ONFH tissues as compared to adjacent normal tissues, 19 of which were downregulated and nine upregulated. Moreover, they found that two CpG sites of miR-210 were hypermethylated in the normal bone tissue as compared to the steroid-induced ONFH tissue, likely causing suppression of miR-210 expression in these tissues. However, once ONFH occurs, the self-reparative mechanism would be switched on and miR-210 is demethylated to upregulate the expression of miR-210, which could activate angiogenesis to promote femoral head healing.

Chao et al. [11] demonstrated that miR-1207-5p was highly expressed in the necrotic femoral head tissue and in the peripheral blood of patients with steroid-induced ONFH. The content of free miR-1207-5p in the serum of the steroid-induced ONFH group was about 2.5 times higher than that of the control group. Interestingly, its expression level was inversely proportional to the Harris Hip score. They further revealed that the regulatory effect of miR-1207-5p on steroid-induced ONFH was related to its effect on epidermal growth factor receptors using rat models.

### 2.2. miRNA Expression Profiles in Serum

Wei and Wei [23] used a miRNA microarray to profile miRNA expression in the serum of patients with steroid-induced ONFH and healthy volunteers in an attempt to identify new biomarkers for diagnosis. They found three miRNAs that were downregulated and nine miRNAs that were upregulated in the serum of the ONFH patients. Moreover, RT-qPCR confirmed a significant increase in the level of miR-10a-5p and a decrease in the level of miR-423-59 in the patient samples. Similarly, Li et al. [16] found 11 differentially expressed miRNAs (two upregulated and nine downregulated) between the ONFH group and healthy control group. Furthermore, 42 differentially expressed miRNAs (14 upregulated and 28 downregulated) were found between the ONFH group and a control group comprising patients with systemic lupus erythematosus (SLE).

Wang et al. [14] screened circulating miRNAs using Illumina-based high-throughput sequencing technology and found 27 circulating miRNAs that were differentially expressed in the serum of patients with steroid-induced ONFH. Among them, the levels of 15 and 12 miRNAs were increased and decreased, respectively, when compared to those in the serum of both SLE patients treated with steroid-based regimens and healthy subjects. The same group of researchers chose six miRNAs with relatively high specificity and sensitivity for further validation by RT-qPCR [15], demonstrating that the circulating levels of miR-10a-5p, miR-99a-5p, and miR-21-5p were all increased in both the steroid-treated SLE patients and rat models regardless of ONFH development. These miRNAs therefore show potential as biomarkers of general steroid exposure. Two common miRNAs, miR-10a-5p and miR-423-59, were found among these studies, which may need more attention for further studies.

### 2.3. miRNA Expression Profiles in Bone Marrow Mesenchymal Stem Cells (BMSCs)

Bian et al. [25] profiled miRNA expression in human BMSCs from subjects treated without or with steroids (10^–7^ or 10^–9^ mol/l dexamethasone) and identified 11 upregulated and six downregulated miRNAs in the steroid-treated group. Zhao et al. [10] found that the expression level of hsa-miR-122-3p in the steroid-induced ONFH group was significantly lower than that of patients with a femoral neck fracture using RT-qPCR. In addition, the expression level of hsa-miR-122-3p in the group at Ficat stage IV was found to be lower than that in the stage III group, suggesting that the expression level of hsa-miR-122-3p in BMSCs may be correlated with the progression of steroid-induced ONFH. Wang et al. [13] profiled the miRNA expression in three patients with steroid-induced ONFH and three patients with femoral neck fracture and identified 17 upregulated and five downregulated miRNAs in the steroid-induced ONFH group. Moreover, in an *in vitro* study, they identified five miRNAs (miR-601, miR-452-3p, miR-647, miR-516b-5p, and miR-127-5p) with decreased levels and one miRNA (miRNA-122-3p) with an increased level during osteogenic differentiation, whereas the changes in the expression levels during adipogenic differentiation were in the opposite direction. These findings demonstrated that these six miRNAs have a close relationship with the differentiation of BMSCs. Notably, both of these studies found miRNA-122-3p as deregulated, suggesting an important role in the development and progression of steroid-induced ONFH.

In another study, miRNAs were identified from BMSCs in a rat model of steroid-induced ONFH using a gene chip. Compared with the control group, 23 miRNAs were identified in the model group, with seven upregulated and 16 downregulated miRNAs [24]. Through bioinformatics analysis, they further showed that the upregulated miRNAs miR-21-3p and miR-652-5p, and the downregulated miRNAs miR-34b-3p, miR-34c-5p, miR-148a-3p, miR-196a-5p, and miR-206-3p were predicted to be involved in osteogenic differentiation.

### 2.4. miRNA Expression Profiles in Osteoblasts

Li et al. [19] profiled six differentially expressed miRNAs in steroid-induced ONFH rat models, with four upregulated miRNAs and two downregulated miRNAs. In particular, upregulated miR-672-5p expression and downregulated miR-146a-5p expression were confirmed via RT-qPCR.

### 2.5. miRNA Expression Profiles in Bone Microvascular Endothelial Cells (BMECs)

Yue et al. [12] profiled the differential miRNA expression from rat femoral head BMECs and identified four differentially expressed miRNAs (two upregulated: miR-132-3p and miR-335 and two downregulated: miR-466b-2-3p and let-7c-1-3p) using qPCR and gene-chip analyses. For miRNA expression profiles in human BMECs, Yu et al. [22] identified five miRNAs consistently upregulated/downregulated by at least 2.5-fold in all eight human BMECs samples tested.

### 2.6. Functions of miRNAs in BMSCs

Gu et al. [21] identified 37 differentially expressed miRNAs in the femoral heads of a steroid-induced ONFH rat model. Among these, miR-27a showed downregulated expression in the ONFH model group, along with upregulated expression of the peroxisome proliferator-activated receptor gamma (PPAR*γ*) gene. The authors further verified experimentally that PPAR*γ* is a target of mir-27a. PPAR*γ* is an adipogenic transcription factor that is expressed in the very early stage of adipogenesis. The other potential target identified was gremlin 1, an antagonist of bone morphogenesis protein-2 (BMP-2), which can specifically bind to BMP-2 and block its function. Similarly, Sun et al. [44] found that miR-548d-5p, which was predicted to bind PPAR*γ* 3′-UTR, was downregulated upon steroid-induced adipogenesis in human BMSCs.

Xie et al. [35] reported that miR-181d was upregulated in patients with steroid-induced ONFH, and its expression level was enhanced in proportion with the increased concentration of steroid use. They further showed that miR-181d could inhibit the differentiation of BMSCs into osteoblasts through inhibiting SMAD3. A similar result was also found in another study, in which steroid treatment could inhibit the proliferation of BMSCs and mir-34a expression, and the effect was promoted by increases in the concentration and the time of exposure [32]. Kang et al. [34] proposed that mir-34a-5p is reciprocally regulated by steroids and improves the proliferation and differentiation of osteoblasts by targeting cyclin D1, CDK4, and CDK6 and JAG1/Notch signaling in mice. Notably, the role of Notch signaling in the osteoblastic differentiation of BMSCs and bone formation has yielded conflicting results. Zha et al. [32] verified the promotive effects of these factors and further indicated Runt-related transcription factor 2 (RUNX2) as a target of mir-34a to regulate osteoblastic differentiation. In addition, Hao et al. [20] found that miR-708 expression is inversely correlated with osteonecrosis and that targeting miR-708 could not only promote osteogenic differentiation *in vitro* but also effectively antagonized the suppression effect of steroids on osteoblast and adipocyte differentiation through increasing SMAD3 expression, which may result in an interaction between SMAD3 and RUNX2, and consequent activation of the transforming growth factor-beta (TGF-*β*) signaling pathway.

**Figure 1:**
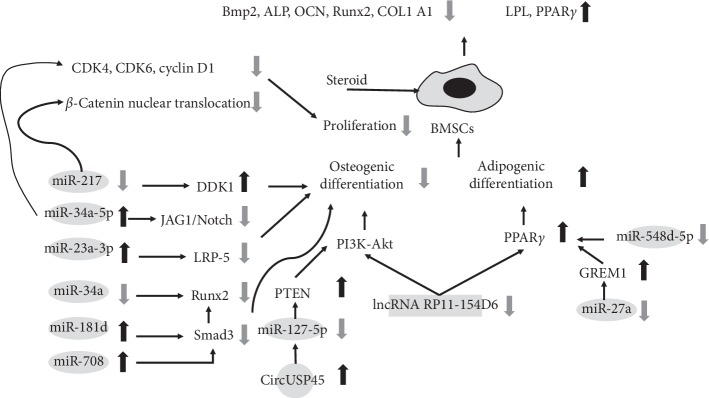
Functions of specific ncRNAs in bone marrow mesenchymal stem cells.

Furthermore, Dai et al. [27] found that miR-217 is notably downregulated in the BMSCs of steroid-induced ONFH patients and that overexpression of miR-217 could significantly promote osteogenic differentiation and proliferation via repressing dickkopf-related protein 1 (DKK1) to promote the nuclear translocation of *β*-catenin. These findings indicate a pivotal role of the miR-217/DKK1/*β*-catenin pathway in the pathological process of steroid-induced ONFH (Figure 1).

### 2.7. Functions of miRNAs in Preosteoblasts and Osteoblasts

Zhang et al. [30] recently demonstrated that miR-206 binds directly and specifically to the promoter sequence of the programmed cell death protein 4 (*PDCD4*) gene. Then, protein repression can further induce the apoptosis of osteoblasts, eventually aggravating steroid-induced ONFH. Furthermore, a previous study demonstrated that the overexpression of miR-206 inhibited the differentiation of osteoblasts via the Wnt/*β*-catenin signaling pathway in a rabbit model [31]. Moreover, Shi et al. [43] found that the level of miR-199a-5p was significantly increased in steroid-treated osteoblasts, which inhibited cell proliferation in differentiating osteoblasts, also by targeting Wnt signaling.

Zhao et al. [36] demonstrated that miR-200a expression activated NF-E2-related factor 2 (NRF2) signaling, which inhibited steroid-induced reactive oxygen species production and osteoblasts death. The miR-200a level was decreased in necrotic femoral head tissues, which was correlated with *KEAP1* mRNA upregulation. Importantly, miR-200a was ineffective in *KEAP1*-silenced human osteoblasts. Fan et al. [42] showed that miR-135b selectively targets the AMP-activated protein kinase (AMPK) phosphatase Ppm1e, whose downregulation induced profound AMPK activation in osteoblastic cells. Significantly, the miR-135b level was increased in human necrotic femoral head tissues, which was correlated with Ppm1e downregulation and AMPK activation. Interestingly, AMPK activation can exert an antioxidant function and protect stressed cells. In addition, AMPK knockdown or mutation abolished this miR-135b-induced cytoprotection against steroid treatment in osteoblastic cells, supporting a critical function of AMPK activation in the actions of miR-135b. Subsequently, they found that miR-25-5p level was increased in patients' necrotic femoral head tissues, targeting and downregulating PKC*ζ* in osteoblastic cells and in turn activating AMPK signaling and protecting cells against the damage induced by steroids [40].

Bai et al. [28] reported that the induction of TGF-*β*/SMAD7 signaling in preosteoblasts may be a potential mechanism by which miR27a regulates steroid-induced ONFH. Upregulation of SMAD7 protein expression enhanced the effects of miR27a overexpression on osteoblastic differentiation, cell proliferation, ALP activity, osteonectin mRNA expression, and SMAD7 protein expression in the mouse osteoblastic cell line MC3T3E1. The accumulation of activated SMAD compounds in the nucleus plays a crucial role in the transmission of TGF-*β* signals from transmembrane receptors to the cell nucleus. They also found that the serum levels of miR27a were decreased in a rat model of ONFH when compared with those in normal controls. In addition, Shi et al. [45] showed that steroids could increase the expression level of receptor activator of nuclear factor B ligand (RANKL) by downregulating miR-17/20a in osteoblasts, which indirectly enhances osteoclastogenesis and bone resorption.

**Figure 2:**
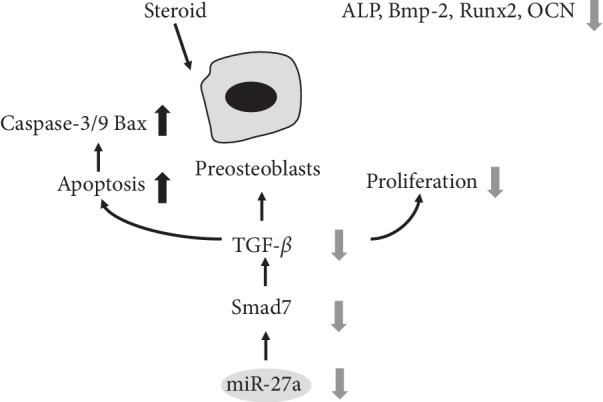
Functions of specific ncRNAs in preosteoblasts.

**Figure 3:**
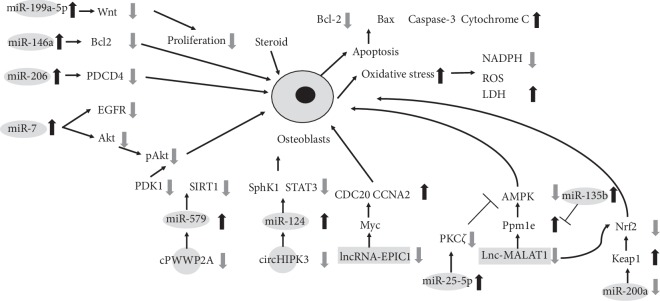
Functions of specific ncRNAs in osteoblasts.

Furthermore, Zhang et al. [37] found that miR-146a regulates the proliferation and apoptosis of murine osteoblastic MC3T3-E1 cells. Dexamethasone-stimulated MC3T3-E1 cells showed upregulation of miR-146a, accompanied by a decreased expression level of the antiapoptotic gene *Bcl2*, leading to increased apoptosis and decreased proliferation of MC3T3-E1 cells; thus, overexpression of miR-146a increased the apoptotic sensitivity of MC3T3-E1 cells to dexamethasone stimulation (Figures 2 and 3).

### 2.8. Functions of miRNAs in Endothelial Cells

**Figure 4:**
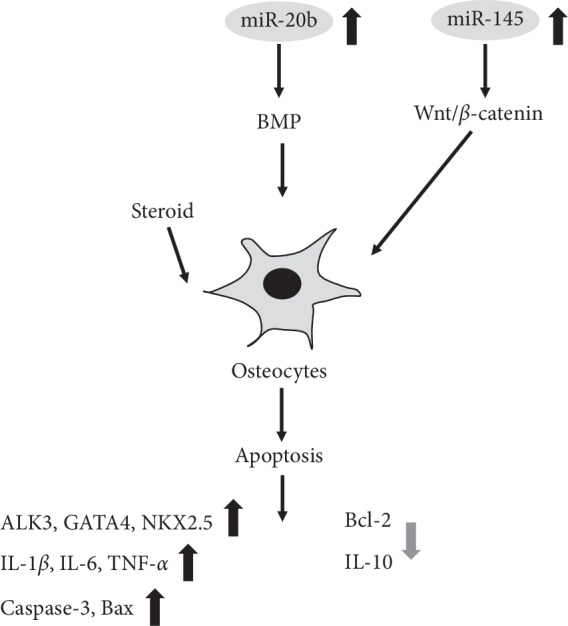
Functions of specific ncRNAs in endothelial cells.

Zha et al. [32] showed that steroid administration suppressed the viability of human umbilical vein endothelial cells and vascular endothelial growth factor (VEGF) secretion in a dose- and time-dependent manner, while the mir-34a expression level was increased. They suggested that the transfection of an mir-34a overexpressing lentivirus led to a direct and acute inhibitory effect on angiogenesis in the early stage of steroid-induced ONFH, but disturbed the normal vascular reparative process in the compensatory phase, which then indirectly aggravated ONFH (Figure 4).

### 2.9. Functions of miRNAs in Animal Models

Wei et al. [29] found that the miR-320 expression level significantly decreased in patients with steroid-induced ONFH, accompanied by increased cytochrome P450 1A2 (CYP1A2) expression and activity. In animal models, luciferase reporter gene detection demonstrated that *Cyp1a2* is the downstream target gene of miR-320, while hemodynamic and microcirculatory analyses indicated that upregulated CYP1A2 expression can greatly promote the occurrence and development of steroid-induced ONFH.

Li and Wang [26] reported that steroid-induced ONFH can significantly increase the expression level of miR-20b in rat bone tissues targeting the BMP signaling pathway. Tian et al. [38] found that miR-145 silencing could increase the expression levels of VEGF and basic fibroblast growth factor to promote angiogenesis in rabbit models. In addition, miR-145 silencing inhibited the apoptosis of bone cells in a steroid-induced ONFH model through the Wnt/*β*-catenin pathway.

Peng et al. [33] showed that miR-34a can inhibit transforming growth factor-beta-induced factor homeobox 2 (Tgif2) and osteoprotegerin (OPG)/RANK/RANKL signals, ultimately reducing the progression of steroid-induced ONFH. OPG, an inhibitor of osteoclast paracrine function, is produced by osteoblasts and combines with RANKL to prevent the interaction of RANKL and RANK so as to suppress the activation of osteoclasts and consequently their differentiation. Zhao et al. [39] also found that miR-145 can improve steroid-induced ONFH in rats by inhibiting the OPG/RANK/RANKL signaling pathway.

**Figure 5:**
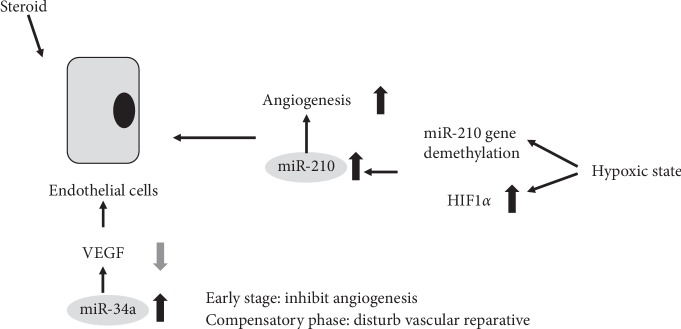
Functions of specific ncRNAs in osteocytes.

In addition, Dong et al. [41] demonstrated that injection of a miR-23a-3p inhibitor could decrease the incidence of osteonecrosis in a rat model. They had previously demonstrated that miR-23a was partially complementary to a site in the 3′-UTR of low-density lipoprotein receptor-related protein 5 (LRP-5), which plays a crucial role in bone formation. Mice lacking LRP-5 exhibit a decrease in bone mass, while LRP-5 activation increases bone mass. The prevalence of osteonecrosis was 18.2% and 75% in the miR-23a-3p-inhibitor and miR-23a-3p mimic groups, respectively (Figure 5). The functions of the miRNAs identified to be associated with steroid-induced ONFH are summarized in Table 2.

## 3. Long Noncoding RNAs (lncRNAs) in Steroid-Induced ONFH

Aberrant gene expression can cause alterations in cell behaviors. As summarized above, there has been extensive research effort on the roles of ncRNAs, especially miRNAs, in regulating BMSCs phenotypes. Other ncRNAs have also been investigated in the regulation of orthopedic disorders such as osteoarthritis and osteoporosis [46, 47]. However, studies focusing on lncRNAs in patients with steroid‐induced ONFH are relatively scarce. lncRNAs are RNA sequences longer than 200 nucleotides that usually display mRNA‐like characteristics such as being 5′‐capped, spliced, and polyadenylated. lncRNAs can alter protein expression and/or functions in several different manners [48], and their association with ONFH is gradually coming to light through studies with patient tissues, along with *in vitro* investigations in BMSCs, BMECs, and osteoblasts.

### 3.1. lncRNA Expression Profiles in ONFH Tissues

Luo et al. [49] compared the lncRNA expression profiles in three patients with steroid-induced ONFH and three patients with femoral neck fracture by microarray, revealing a total of 1179 upregulated and 3214 downregulated lncRNAs in ONFH tissues. The upregulated lncRNAs (ENST00000532068, NR 027293, NR 046211, and T278056) and the downregulated lncRNAs (T318776, NR 038891, ENST00000565178, and ENST00000445662) in the ONFH group were confirmed by RT-qPCR.

### 3.2. lncRNA Expression Profiles in BMSCs

In a recent study, a total of 1147 differentially expressed lncRNAs were identified in BMSCs isolated from patients with steroid-induced ONFH [5], including the confirmed downregulated expression of the lncRNA RP11‐154D6 by qRT-PCR. In another study using steroid-induced ONFH mouse models, the lncRNA RP11‐154D6 was shown to exert its function through interactions with miR‐30a [24]. However, no significant relationship was found between the expression levels of lncRNA RP11‐154D6 and miR‐30a‐5p, indicating that lncRNA RP11‐154D6 may regulate differentiation through mechanisms other than targeting miR‐30a‐5p. Wang et al. [50] also investigated the lncRNA expression profile of BMSCs from patients with steroid-induced ONFH and identified 3720 lncRNAs and 2775 mRNAs that were differentially expressed. In addition, they explored the mechanisms of steroid-induced ONFH using bioinformatics analysis. Their results suggested that the lncRNA expression profiles were linked to the irregular adipogenic and osteogenic differentiation of BMSCs during steroid-induced ONFH progression.

### 3.3. lncRNA Expression Profiles in BMECs

Yu et al. [22] examined the differential expression and hierarchical clustering of lncRNAs in BMECs treated with or without glucocorticoids. They found 73 upregulated lncRNAs and 166 downregulated lncRNAs, 107 of which were significantly correlated with the expression of 172 mRNAs induced by the steroid. A summary of the expression profiles in steroid-induced ONFH is provided in Table 3.

### 3.4. lncRNAs in Osteoblasts

Zhang et al. [51] found that lncRNA-EPIC1 expression protects human osteoblasts from damage induced by steroids via regulation of Myc. Although steroid treatment showed no effect on Myc expression itself, it did increase the expression levels of Myc target genes (*CDC20* and *CCNA2*) possibly due to upregulation of Lnc-EPIC1. Fan et al. [52] found that Lnc-MALAT1 expression was downregulated by dexamethasone treatment in osteoblastic cell lines and primary human osteoblasts. Importantly, forced overexpression of Lnc-MALAT1 could protect human osteoblasts from dexamethasone-induced injury and inhibited oxidative stress via activation of AMPK and NRF2 signaling (Figure 3). Interestingly, Lnc-MALAT1 was also found an important function in BMSCs in another study [50]. Hence, it may play a wider role in different cells. The functions of the lncRNAs identified to be associated with steroid-induced ONFH are summarized in Table 4.

## 4. Circular RNAs (circRNAs) in Steroid-Induced ONFH

CircRNAs are relatively new biomarkers among ncRNA clusters, which play important roles in metabolic activities such as immune surveillance, cell cycle regulation, and human embryonic stem cell pluripotency [53]. Recently, there has been increasing interest in circRNAs because of their diverse biological functions in bone remodeling. However, research on the roles of circRNAs in steroid-induced ONFH is at an even earlier stage than the other types of ncRNAs. Nevertheless, some recent *in vitro* studies can provide some insight and guidance for further focus of this intriguing ncRNA population as potential biomarkers for steroid-induced ONFH.

### 4.1. CircRNAs in BMSCs

Kuang et al. [54] reported the relationship between circUSP45 in steroid-induced ONFH and bone metabolism. The authors found that circUSP34 regulates osteogenesis and proliferation of BMSCs by sponging miR-127-5p and targeting the PTEN/AKT pathway. To verify the function of circUSP45 *in vivo*, the authors used a steroid-induced ONFH rat model, demonstrating that inhibiting circUSP45 improved bone mass (Figure 1).

### 4.2. CircRNAs in Osteoblasts

Zhu et al. [6] found that circHIPIK3 downregulation was vital in mediating the dexamethasone-induced cytotoxicity in human osteoblasts. Dexamethasone treatment to human osteoblasts induced a reduction in the level of circHIPK3 accompanied by the accumulation of miR-124 and downregulation of the miR-124 prosurvival targets *SPHK1* and *STAT3*. Conversely, lentivirus-mediated ectopic overexpression of circHIPIK3 attenuated the dexamethasone-induced apoptosis and programmed necrosis in human osteoblasts. A similar study showed that steroid treatment in human osteoblasts induced *cPWWP2A* downregulation, causing miR-579 accumulation but depletion of its targets (*SIRT1* and *PDK1*), eventually leading to cell apoptosis and programmed necrosis [55]. These results indicate that targeting the circRNA-miR axis could be a novel strategy to protect against steroid-induced damage to human osteoblasts (Figure 3). The functions of the circRNAs reported to be associated with steroid-induced ONFH are summarized in Table 5.

## 5. Conclusion and Prospects of the Roles and Applications of ncRNAs in ONFH

There is now strong evidence indicating an important role of ncRNAs in the development and progression of steroid-induced ONFH. To better understand the functions of ncRNAs in steroid-induced ONFH, future studies should focus on the molecular mechanisms by which the altered expression of ncRNAs contributes to steroid-induced ONFH, particularly through identification of the ncRNA regulation network and the interaction between ncRNAs and DNA. By ncRNA profiling, several studies identified differentially abundant ncRNAs in serums of steroid-induced ONFH patients such as miR-10a-5p, miR-423-59, miR-99a-5p, and miR-21-5p, suggesting their potential use as diagnostic markers. These may further improve the diagnostic efficiency combined with image examination.

Notably, the same ncRNAs may play different roles in different cell types. Thus, future studies may need to address this problem using single-cell transcriptomics or other techniques. Although the clinical value of ncRNAs is only beginning to surface, the available data already highlight the prospective use of ncRNA-based therapies in the treatment or diagnosis of steroid-induced ONFH.

## Figures and Tables

**Table 1:** miRNAs expression profiles in steroid-induced ONFH.

No.	Evaluation method	Sample	Dysregulation (up)	Dysregulation (down)	References
1	qRT-PCR	Human BMSCs	—	hsa-miR-122-3p	[10]
2	qRT-PCR	Human and rat ONFH tissue and serum	miR-1207-5p	—	[11]
3	Microarray qRT-PCR	Rat BMECs	2 miRNAs miR-132-3p, miR-335	2 miRNAs miR-466b-2-3p, let-7c-1-3p	[12]
4	Microarray qRT-PCR	Human BMSCs	17 miRNAs hsa-miR-601, hsa-miR-452-3p, hsa-miR-647, and hsa-miR-516b-5p	5 miRNAs hsa-miR-122-3p	[13]
5	Microarray qRT-PCR	Human and rat serum	15 miRNAs miR-10a-5p, miR-99a-5p, miR-21-5p	12 miRNAs	[14, 15]
6	Microarray	Human serum	2 miRNAs	9 miRNAs	[16]
7	Microarray qRT-PCR	Human ONFH tissue	8 miRNAs	2 miRNAs has-miR-195-5p	[17]
8	Microarray qRT-PCR	Human ONFH tissue	9 miRNAs miR-210	19 miRNAs	[18]
9	Microarray qRT-PCR	Rat osteoblasts	4 miRNAs miR-672-5p	2 miRNAs miR-146a-5p	[19]
10	Microarray qRT-PCR	Human BMSCs	2 miRNAs hsa-mir-483-5p, hsa-mir-708	6 miRNAs hsa-mir-92a-1, hsa-mir-25	[20]
11	Microarray qRT-PCR	Rat BMSCs	9 upregulated miR-27a	28 downregulated miR-182	[21]
12	Microarray qRT-PCR	Human BMECs	5 up hsa-miR-339-5p	11 down hsa-miR-100-3p hsa-miR-222-5p hsa-miR-23b-5p hsa-miR-933	[22]
13	Microarray qRT-PCR	Human serum	9 up miR-423-5p	3 down miR-10a-5p	[23]
14	Microarray qRT-PCR	Mice BMSCs	23 miRNAs miR-21-3p miR-652-5p	16 miRNAs miR-34b-3p, miR-34c-5p, miR-148a-3p, miR-196a-5p, miR-206-3p	[24]
15	Microarray qRT-PCR	Human BMSCs	11 miRNAs hsa-miR-4289 hsa-miR-378g hsa-miR-378f hsa-miR-378d hsa-miR-196b-5p hsa-miR-196a-5p hsa-miR-16-5p hsa-miR-1268b hsa-miR-1268a hsa-miR-107 hsa-miR-103a-3p	6 miRNAs hsa-miR-4634 hsa-miR-4448 hsa-miR-378i hsa-miR-378h hsa-miR-378a-3p hsa-miR-24-3p	[25]

*Note*. miRNAs, microRNAs; ONFH, osteonecrosis of the femoral head; qRT-PCR, quantitative real-time polymerase chain reaction; BMSCs, bone marrow mesenchymal stem cells; BMECs, bone microvascular endothelial cells.

**Table 2:** Functions of the miRNAs in steroid-induced ONFH.

Evaluated miRNA(s)	Study type	Involved cells/in vivo model/clinical samples	Target(s)	Pathway	Functions	Dysregulation (up/down)	Species	References
miR-20b	In vivo	Osteocytes and rat	BMP	BMP	Block the angiogenesis in bone tissues, promoting osteocyte apoptosis	Up	Rat	[26]

miR-7	In vitro	Osteoblasts	EGFR	EGFR/Akt	Promote osteoblast death	Up	Human	[56]

miR-217	In vitro	BMSCs	DKK1	—	Promote cell proliferation and osteogenic differentiation	Down	Human	[27]

miR-27a	In vivo and vitro	Preosteoblasts and rat	TGF-*β* and Smad7	TGF-*β*/Smad7	Promote osteogenic differentiation and increase proliferation	Down	Human and rat	[28]
	In vivo and vitro	mBMSCs and rat	PPAR*γ* and GREM1	PPAR*γ*	Suppress adipogenesis and enhance osteogenesis	Down	Rat	[21]

miR-320	In vivo and in vitro	Human ONFH tissue and rat	CYP1A2	—	Inhibit the occurrence and development of ONFH	Down	Human and rat	[29]

miR-206	In vitro	Osteoblasts	PDCD4	—	Decrease cell viability and proliferation, while apoptosis was induced	Up	Human	[30]
	In vivo and in vitro	Osteoblasts and rabbit	Cx43	Wnt/*β*-catenin	Inhibit the differentiation of osteoblasts	Up	Rabbit	[31]

mir-34a	In vivo and in vitro	mBMSCs, HUVECs, and rat	Runx2	—	BMSCs: Promote osteogenic differentiation and increase proliferation HUVECs: acute inhibitory effect on angiogenesis in the early stage of steroid-induced ONFH, but it disturbed the normal vascular reparative process in the compensatory phase, which then indirectly aggravates ONFH.	Down in mBMSCs, and up in HUVECs	Human and rat	[32]
	In vivo	Rat serum	Tgif2	OPG/RANK/RANKL	Alleviate ONFH	Down	Rat	[33]
	In vitro and in vivo	mBMSCs and mice	CDK4, CDK6, and cyclin D1	JAG1/Notch	Inhibit BMSCs proliferation and osteoblastic differentiation	Up	Mice	[34]

miR-181d	In vitro	hBMSCs	Smad3	Smad3	Inhibit osteobalsitc differentiation of BMSCs	Up	Human	[35]

miR-200a	In vitro	Osteoblasts	Keap1/Nrf2	Nrf2	Protect osteoblasts from dex	Down	Human	[36]

miR-146a	In vitro	Murine osteoblasts	Bcl2	Bcl2	Inhibits proliferation and induce apoptosis	Up	Murine	[37]
	In vivo	Rabbit	Wnt	Wnt/*β*-catenin	Promote angiogenesis; inhibited the apoptosis of bone cells	Up	Rabbits	[38]

miR-145	In vivo and in vitro	Human THP-1 cells and rat	OPG	OPG/(RANKL)/RANK	Inhibit osteoclast differentiation and prevent excessive bone resorption	Down	Human and rat	[39]

miR-25-5p	In vitro	Osteoblasts	PKC*ζ*	AMPK	Protect osteoblasts from steroid	Up	Human	[40]

miR-23a-3p	In vivo and in vitro	mBMSCs and rat	LRP5	—	Inhibit osteogenic differentiation	Up	Rat	[41]

miR-708	In vivo and in vitro	hBMSCs	Smad3	Smad3	Inhibit osteogenic differentiation and enhance adipogenesis differentiation	Up	Human	[20]

miR-210	In vivo and invitro	HUVECs and rat	—	—	activate the angiogenesis	Up	Human and rat	[18]

miR-135b	In vitro	Osteoblastic	Ppm1e	AMPK	Protects osteoblasts from steroid	Up	Human	[42]

mir-199a-5p	In vivo and intro	Osteoblasts and rat	Wnt	FZD4, WNT2	Inhibit cell proliferation in differentiating osteoblasts	Up	Human and rat	[43]

miR-548d-5p	In vitro	hBMSCs	PPAR*γ*	PPAR*γ*	Suppress steroid-induced adipogenic differentiation of and enhance osteogenic potential.	Down	Human	[44]

miR-17/20a	In vivo and vitro	Osteoblasts and rat	RANKL	RANKL	Inhibit steroid-induced osteoclast differentiation	Down	Human and rat	[45]

*Note*. miRNAs, microRNAs; ONFH, osteonecrosis of the femoral head; BMSCs, bone marrow mesenchymal stem cells; GREM1, gremlin 1; BMP, bone morphogenesis protein; EGFR, epidermal growth factor receptor; DKK1, dickkopf-related protein 1; TGF-*β*, transforming growth factor-*β*; PPAR*γ*, peroxisome proliferator-activated receptor gamma; Runx2, Runt-related transcription factor 2; CYP1A2, cytochrome P450 1A2; PDCD4, programmed cell death protein 4; Cx43, Connexin43; HUVECs, human umbilical vein endothelial cells; Tgif2, transforming growth factor-beta-induced factor homeobox 2; OPG, osteoprotegerin; RANK, Receptor activator of nuclear factor B; RANKL, receptor activator of nuclear factor B ligand; Nrf2, NF-E2-related factor 2; Bcl2, B-cell lymphoma-2; PKC*ζ*, protein kinase C *ζ*; AMPK, AMP-activated protein kinase; LRP-5, low-density lipoprotein receptor-related protein 5.

**Table 3:** lncRNAs expression profiles in steroid-induced ONFH.

No.	Evaluation method	Sample	Dysregulation (up)	Dysregulation (down)	References
1	Microarray qRT-PCR	Human BMSCs	181 lncRNAs	391 lncRNAs lncRNA RP11‐154D6	[5]

2	Microarray qRT-PCR	Human ONFH tissue	1179 lncRNAs lncRNA NR 027293	3214 lncRNAs lncRNAs ENST00000565178, NR 038891, T318776	[49]

3	Microarray qRT-PCR	Human BMSCs	1878 lncRNAs OGFR-AS1, LOC100505817, HOTAIR, RP1-67K17.3, CTD-2006O16.2	1842 lncRNAs RP1-193H18.2, XXBAC-BPGBPG55C20.3, MALAT1, CTD-3080F16.3, and RUNX1-IT1	[50]

4	Microarray qRT-PCR	Human BMECs	73 up lncRNAp4493	166 down lncRNAp19376	[22]

*Note*. lncRNAs, long noncoding RNAs; ONFH, osteonecrosis of the femoral head; qRT-PCR, quantitative real-time polymerase chain reaction; BMSCs, bone marrow mesenchymal stem cells; BMECs, bone microvascular endothelial cells.

**Table 4:** Functions of the lncRNAs in steroid-induced ONFH.

Evaluated lncRNA (s)	Study type	Involved cells	Target (s)	Pathway	Functions	Dysregulation (up/down)	Species	References
lncRNA RP11‐154D6	In vitro	BMSCs	—	—	Promote osteogenic differentiation and inhibit adipogenic differentiation	Down	Human	[5]

LncRNA EPIC1	In vitro	Osteoblasts	Myc (CDC20 and CCNA2)	—	Protect osteoblasts from steroid	Up	Human	[51]

Lnc-MALAT1	In vitro	Osteoblasts	Ppm1e	AMPK; Nrf2	Protect osteoblasts from steroid and attenuate oxidative stress	Down	Human	[52]

Note: LncRNAs, long noncoding RNAs; ONFH, osteonecrosis of the femoral head; BMSCs, bone marrow mesenchymal stem cells.

**Table 5:** Functions of the CircRNAs in steroid-induced ONFH.

Evaluated circRNA(s)	Study type	Involved cells/in vivo model	Target(s)	Pathway	Functions	Dysregulation (up/down)	Species	References
circHIPK3 (circ_0000284)	In vitro	Osteoblasts	miR-124	SphK1 and STAT3	Suppress steroid-induced apoptosis and programmed necrosis of osteoblasts	Down	Human	[6]

circUSP45	In vivo and in vitro	hBMSCs and rats	miR-127-5p	PTEN/AKT	Decrease osteogenic gene expression and inhibit the proliferation of hBMSCs	Up	Human and rat	[54]

cPWWP2A	In vitro	Osteoblasts	miR-579	SIRT1 and PDK1	Suppress steroid-induced apoptosis and programmed necrosis of osteoblasts	Down	Human	[55]

*Note*. CircRNAs, circular RNAs; ONFH, osteonecrosis of the femoral head; BMSCs, bone marrow mesenchymal stem cells.

## Abbreviations

AMPK: AMP-activated protein kinase
BMECs: Bone microvascular endothelial cells
BCL2: B-cell lymphoma-2
BMP: Bone morphogenic protein
BMSCs: Bone marrow mesenchymal cells
CYP1A2: Cytochrome P450 1A2
DKK1: Dickkopf-related protein 1
LRP-5: Low-density lipoprotein receptor-related protein 5
miRNA: MicroRNA
ncRNA: Noncoding RNA
NRF2: NF-E2-related factor 2
ONFH: Osteonecrosis of the femoral head
OPG: Osteoprotegerin
PPAR*γ*: Peroxisome proliferator-activated receptor gamma
RANKL: Receptor activator of nuclear factor B ligand
RT-qPCR: Reverse transcription-quantitative polymerase chain reaction
RUNX2: Runt-related transcription factor 2
SLE: Systemic lupus erythematosus
TGF-*β*: Transforming growth factor-beta
UTR: Untranslated region
VEGF: Vascular endothelial growth factor
